# Complete genome sequence of *Lactiplantibacillus brownii* strain MH-1 isolated from Japanese traditional homemade pickled Chinese cabbage

**DOI:** 10.1128/mra.01064-24

**Published:** 2024-12-20

**Authors:** Mina Hashimoto, Yoshihiko Tanimoto, Yen Lin Chen, Takayuki Wada, Eriko Kage-Nakadai

**Affiliations:** 1 Graduate School of Human Life and Ecology, Osaka Metropolitan University 12936 , Osaka, Japan; 2 Institute for Life and Medical Sciences, Kyoto University 12918 , Kyoto, Japan; 3 Osaka International Research Center for Infectious Diseases, Osaka Metropolitan University 12936 , Osaka, Japan; Queens College Department of Biology, Queens, New York, USA

**Keywords:** *Lactiplantibacillus brownii*, complete genome sequence

## Abstract

*Lactiplantibacillus brownii* was recently reported as a novel species. Here, we present the whole-genome sequence of *Lactiplantibacillus brownii* strain MH-1 isolated from homemade pickled Chinese cabbage in Japan. These genomic data have the potential to help clarify the role of *Lactiplantibacillus* species in fermented foods.

## ANNOUNCEMENT

*Lactiplantibacillus brownii* was isolated from sauerkraut in Russia and reported as a novel species (1). *Lactiplantibacillus* species are found in diverse environments and fermented foods, including vegetables, fish, dairy products, sourdough, and meats (2). The traditional Japanese process of preserving food by fermenting it in rice bran, called nukazuke, is one of their sources (3, 4). *L. brownii* strain MH-1 was isolated from homemade pickled Chinese cabbage made in Hiroshima Prefecture, Japan.

Here, we announce the complete genome sequence of *Lactiplantibacillus brownii* strain MH-1. The diluted pickle juice was plated on MRS agar (Difco BD, Sparks, MD, USA) supplemented with 1% calcium carbonate. Plates were incubated for 48 h at 30°C anaerobically. A single colony was cultured in MRS Broth (Difco BD) for 24 h in static culture, and DNA was isolated using the NucleoSpin Microbial DNA (Macherey and Nagel, Düren, Germany) and 2.0 µg was obtained from 1 mL of broth. Short-read sequencing was performed by preparing Illumina sequencing libraries using the QIAseq FX DNA Library Kit (Qiagen, Germantown, MD, USA), with an initial amount of 80 ng DNA and a MiSeq system with v3 chemistry (2 × 300 bp, 600 cycles) following the instructions of the manufacturer (Illumina, San Diego, CA, USA), resulting in a total of 2,574,412 raw short reads (588,869,869 bp). Long-read sequencing was conducted by preparing Nanopore sequencing libraries with 1 µg high-molecular-weight DNA without DNA shearing and size selection using the Ligation Sequencing Kit (SQK-LSK109, Oxford Nanopore Technology [ONT], Lexington, MA, USA). The libraries were sequenced in FLO-MIN-106D flow cell (ONT) and base-called (high accuracy base calling, Guppy 6.3.9) on Mk1B platform. After base calling by MinKNOW 22.10.10, a total of 97,929 raw long reads (1,001,258,972  bp [*N*_50_, 13,059 bp]) were obtained. The sequence quality was validated with the “stats” command of Seqkit version 2.0.0 (5). Using the long reads, genome assembly was directly (without trimming) performed by Flye version 2.9.1-b1780 (6) and Unicycler version 0.4.3 (7). Three complete circular contigs were obtained, confirmed by both assembly results showing that no misassemblies were found, and were polished using short reads with Pilon version 1.24 (8) three times. They were then reoriented to dnaA and annotated with DFAST version 1.4.0 (9). Default parameters were used for all software.

The completed genome of MH-1 consists of one circular chromosome (3,044,547 bp, 43.75% GC) and two circular plasmids, pMH-1L (41,721 bp, 40.01% GC) and pMH-1S (37,225 bp, 39.99% GC), with an overall sequencing coverage of ~303×. Genome and plasmid sequence details can be found in Table 1.

**TABLE 1:** Genome and plasmid sequence details of strain MH-1

Feature	*Lactiplantibacillus brownii* strain MH-1	pMH-1L	pMH-1S
Size (bp)	3,044,547	41,721	37,225
GC content (%)	43.75	40.01	39.99
Genes	2,907	44	44
CDS	2,827	44	44
Functional proteins	2,061	26	22
Hypothetical proteins	766	18	22
rRNAs (5S, 16S, 23S)	16	0	0
tRNAs	63	0	0
tmRNAs	1	0	0

**Fig 1:**
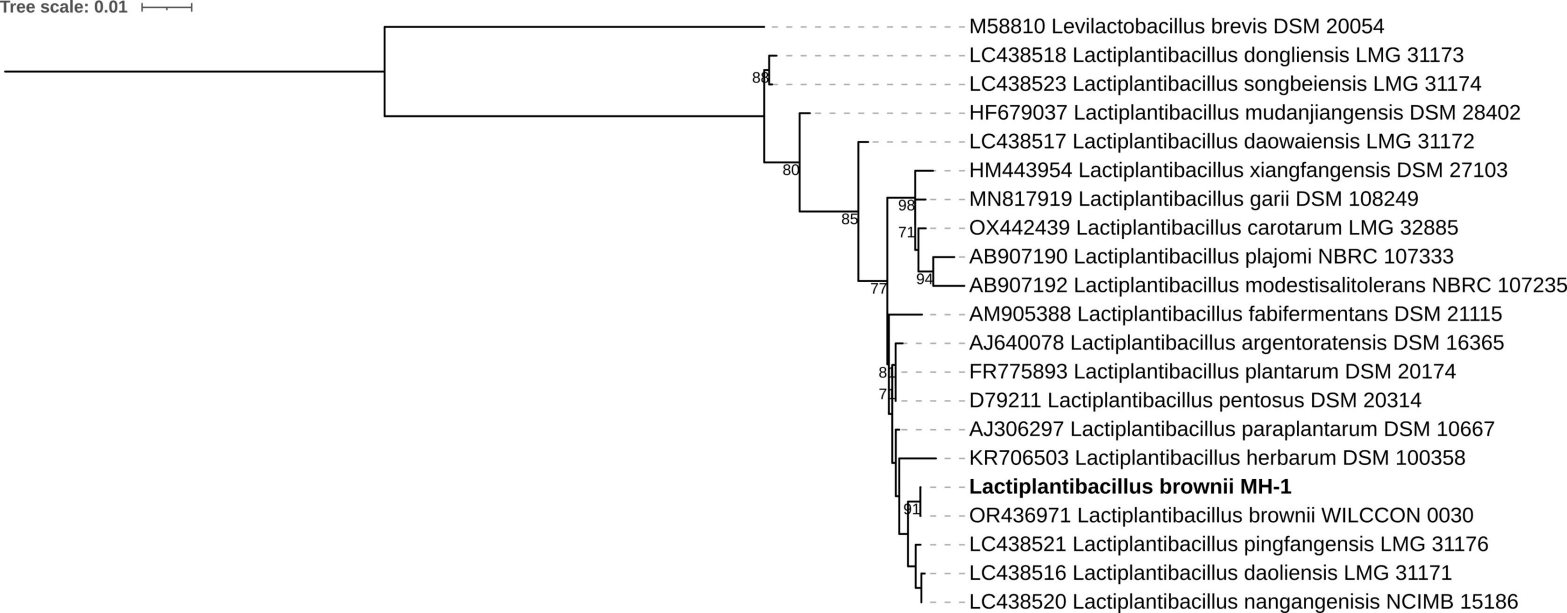
Phylogenetic tree of *Lactiplantibacillus* 16S gene. The tree was constructed using IQ-TREE version 2.2.6 and is based on the alignment of the nucleic acid sequence of 16S gene. TPM3 + R2 model was selected. Members of *Lactiplantibacillus* spp. were selected by referring to the previous report (13). *Levilactobacillus brevis* DSM 20054 was set as an outgroup. The numbers shown at the branches represent the bootstrap support values (>70) obtained from 1,000 bootstrap replicates. The scale bar represents the number of nucleic acid substitutions per site.

Multiple alignments of DNA sequences were performed using MAFFT version 7.520 (10). A phylogenetic tree was constructed with IQ-TREE version 2.2.6 (11). The phylogenetic tree using complete 16S genes indicated that the closest matching species of MH-1 was *L. brownii* WILCCON 0030 (Fig. 1). Average nucleotide identity was 99.86% between WILCCON 0030 and MH-1 using FastANI version 1.1 (12).

## Data Availability

The complete genome sequences for Lactiplantibacillus brownii strain MH-1 were deposited in DDBJ/ENA/GenBank under the accession numbers AP027463 (Chromosome), AP027464 (pMH-1L), and AP027465 (pMH-1S), respectively. The read archives have been deposited in the DDBJ Sequence Read Archive (DRA) under the accession numbers DRR584458-DRR584459.
